# Invasive Candida Infections in Patients With Haematological Malignancies and Hematopoietic Stem Cell Transplant Recipients: Current Epidemiology and Therapeutic Options.

**DOI:** 10.4084/MJHID.2011.013

**Published:** 2011-03-15

**Authors:** Corrado Girmenia, Erica Finolezzi, Vincenzo Federico, Michelina Santopietro, Salvatore Perrone

**Affiliations:** Dipartimento di Ematologia, Oncologia, Anatomia Patologica e Medicina Rigenerativa, Azienda Policlinico Umberto I, Rome, Italy

## Abstract

In the last decades, the global epidemiological impact of invasive candidiasis (IC) in patients with hematologic malignancies (HM) and in hematopoietic stem cell transplant (HSCT) recipients has decreased and the incidence of invasive aspergillosis exceeded that of Candida infections. The use of prevention strategies, first of all antifungal prophylaxis with triazoles, contributed to the reduction of IC in these populations as demonstrated by several epidemiological studies. However, relatively little is known about the current epidemiological patterns of IC in HM and HSCT populations, because recent epidemiological data almost exclusively derive from retrospective experiences and few prospective data are available. Several prospective, controlled studies in the prophylaxis of invasive fungal diseases have been conducted in both the HM and HSCT setting. On the contrary, most of the prospective controlled trials that demonstrated the efficacy of the antifungal drugs echinocandins and voriconazole in the treatment of candidemia and invasive candidiasis mainly involved patients with underlying conditions other than HM or HSCT. For these reasons, international guidelines provided specific indications for the prophylaxis strategies in HM and HSCT patients, whereas the recommendations on therapy of documented Candida infections are based on the results observed in the general population and should be considered with caution.

## Introduction:

Invasive candidiasis (IC) is a major cause of morbidity and mortality in the health care setting. Historically, before the availability of azole antifungal agents for prophylaxis, invasive infections caused by *Candida* species were common in cancer patients, particularly in those affected by hematologic malignancies (HM), and in hematopoietic stem cell transplantation (HSCT) recipients.^1^–^9^ Prolonged neutropenia related to the underlying malignancy and to its intensive treatment represented the main risk factor for this and other IFDs. In the last decades, the overall incidence of IC has significantly increased particularly in the intensive care unit and surgery settings as a consequence of the large use of invasive procedures, intravenous catheters, and intravenous hyperalimentation, all of which are risk factors for candidemia . The importance of these procedures as risk factors for candidemia has been observed also in HM and HSCT patients,^10^,^11^ however, the global epidemiological impact of IC in such populations decreased and the incidence of invasive aspergillosis (IA), exceeded that of Candida infections. The use of prevention strategies, first of all antifungal prophylaxis with triazoles, contributed to the reduction of these infection in cancer patients as demonstrated by several epidemiological studies.^9^,^12^–^14^ On the other hand IC continues to be associated to a high mortality rate in these populations despite the introduction of additional antifungal agents.^15^–^17^

IC encompasses a large variety of diseases in immunocompromised patients, such as bloodstream infection, chronic hepatosplenic candidiasis, endocarditis, osteomyelitis, endophthalmytis and urinary tract infections, however, due to the difficulties in diagnosing invasive infections in sites other than blood, conventionally epidemiological and therapeutic studies on IC almost exclusively include patients with candidemia.

Herein, we will summarize epidemiological and therapeutic aspects of candidemia in HM patients and HSCT recipients as reported in the literature of the last few years with particular attention to the impact of antifungal prophylaxis and therapeutic options with new antifungal drugs.

## Epidemiology:

Relatively little is known about the current epidemiological patterns of IC in HM and HSCT populations. Despite an increasing attention to the clinical and therapeutic aspects of candidemia in the heath care setting, there are few recent large studies on the incidence, microbiologic characteristics, resistance patterns, and clinical outcome of this IFD focused on such populations. Recent epidemiological data almost exclusively derive from retrospective experiences and few prospective data are available.^13^,^14^,^18^–^24^

A nationwide inpatient data base for adult and pediatric patients in the United States showed that in 2000 candidemia was diagnosed in an estimated 1118 hospital admissions of pediatric patients and 8949 hospital admissions of adult patients.^18^ Leukemia or lymphoma was the underling condition in 8% of pediatric patients and 4 % of adult patients. HSCT was the inpatient procedure in 2% of pediatric patients and 0.4% of adult patients.

Very recent clinical data from a general population of patients with candidemia were extracted from the Prospective Antifungal Therapy (PATH) Alliance database, a comprehensive North American registry that collects information regarding IFDs.^19^ Contemporary epidemiology and outcomes of candidemia in multiple centers was evaluated in a total of 2019 patients, enrolled from 2004 through 2008. Data regarding the candidemia episodes were analyzed, including the specific fungal species and patient survival at 12 weeks after diagnosis. Overall, general medicine (66%), surgical non-transplant (33%) and solid tumours (17%) were the populations more represented whereas 9.8% and 2.9% of the candidemia episodes were documented in patients with HM and in HSCT recipients, respectively. Non-albicans Candida species accounted for the large majority of isolates (72.6% in HM and 77.6% in HSCT). This important study on candidemia unfortunately does not offer specific information on prognostic factors, antifungal therapy and outcome in patients with HM but in a further paper some data on HSCT recipients were specifically analyzed.^20^

All these large surveys are able to show that HM and HSCT no more represent the most frequent underlying conditions in patients with candidemia. However, these experiences were not primarily designed to evaluate risk and prognostic factors of IC in subgroups of patients such as those affected by HM or submitted to HSCT.

### Epidemiology in non transplant patients with haematological malignancies:

No prospective multicenter studies and only few retrospective experiences on IFDs in non transplant patients with HM have been published in the last years. Herein, we will summarize the data derived from 3 recent large studies focused in the epidemiology of IC in the HM setting.^14^,^21^,^22^

The largest epidemiological data on IFDs, including IC, in patients with HM derive from a retrospective, muticenter study from Italian hematologic centers published in 2004.^21^ The survey was conducted between January 1999 and December 2003, before the introduction of the new triazoles (voriconazole and posaconazole) and echinocandins (caspofungin, micafungin and anidulafungin). During the study period, 11,802 patients with newly diagnosed hematologic malignancies were admitted to 18 participating centers for treatment other than HSCT. The overall incidence of IFD was 4.6% and the incidence of IC accounted for only 1.5% of patients. The diagnosis was based on blood cultures in all cases. More than 80% of cases of Candida infection were documented in patients with acute leukaemia and the incidence in these patients accounted for 3.5%. The attributable mortality rate was 33% and ranged from 19% in non Hodgkin lymphoma to 36% in acute lymphoid leukemia. Species-specific attributable mortality rate ranged from 6% for infections caused by *C. parapsilosis* to 54% for those due to *C. tropicalis.*

Important epidemiological data on IFD in cancer patients historically derive from the University of Texas MD Anderson Cancer Center (MDACC) . A retrospective, study of candidemia in unselected hematologic cancer and HSCT patients.has been conducted at that institution during the period 2001–2007.^22^ The relevance of this study is also related to the introduction of new antifungal agents such as echinocandins and voriconazole during that time. Overall 173 episodes of candidemia were detected in 170 patients with HM. The incidence of candidemia per 100,000 inpatient days increased from 13.9 episodes in 2001 to 23.2 in 2004, although a slight decrease was observed during the last 2 years of the study (19.2 in 2006). These rates are higher than those reported from multi-institutional European surveys (0.26 to 0.73 per 10,000 inpatient days)^25^,^26^ and United States-based surveillance studies (1.5 cases per 10,000 inpatient days).^27^ Probably the higher incidence observed at the MDACC may be justified by the high immunosuppression in their population and by the particular attention of such institution in the epidemiological control of infectious complications. Most patients (72%) developed breakthrough candidemia while receiving antifungal agents in prophylaxis or treatment of other IFDs. Most infections (76%) were caused by non-albicans Candida species. The shift in the distribution of candidemia-associated Candida species to non-albicans species has been generally attributed to the widespread use of fluconazole, however, in this study, only 29% of patients were receiving fluconazole or itraconazole at the onset of candidemia therefore the non-albicans Candida spp. did not seem to be secondary to the use of triazoles prophylaxis. Furthermore, in vitro susceptibility pattern of Candida isolates in this study demonstrated a stable susceptibility to triazoles compared to previous periods at the same center, in fact the cumulative rate of resistance and dose-dependent susceptibility to triazoles was 29% for fluconazole and 52% for itraconazole. Again, In vitro resistance to amphotericin B and to the antifungal agents introduced into clinical practice after 2000 was uncommon. In fact, only 4%,8%, 6%, and 5% of the isolates exhibited MIC values > 1 mg/L with amphotericin B, voriconazole, posaconazole, and caspofungin.

Crude and attributable mortality rates have remained persistently high despite the introduction of new antifungal agents, and similar to those reported in older studies in haematological patients with candidemia. At day 30 after the documentation of candidemia, the overall mortality rate and attributable mortality rate was 38% and 19%, respectively. Most of deaths (84%) attributed to candidemia occurred by Day 14. The Candida species associated with the highest crude mortality rates at day 30 was C. glabrata (55%), followed by C. krusei (50%) and C.albicans (40%). A multivariate analysis showed that a high fungal burden, measured by quantitative blood cultures in the peripheral blood, and delay or lack of neutrophil recovery were independent predictors of a high early mortality rate. Consistent with a previous study in cancer patients that showed that catheter removal <72 hours after onset improved response to antifungal therapy in patients with CVC related candidemia,^28^ CVC removal within 3 days had a positive effect on early mortality rates.

The authors concluded that the introduction of new antifungal agents and reduction of fluconazole use into clinical practice at their institution did not reduce the incidence of candidemia or the predominance of non-albicans Candida spp. among patients with HM. Furthermore, the use of newer antifungal treatments and association therapies did not reduce the overall and Candida-attributable mortality rates. Probably, the underlying hematologic malignancy, severity of immunosuppression, and presence of indwelling devices represent determinant factors for the outcome more than any antifungal therapy.

A contemporary, nationwide study of candidaemia was conducted in Australia over a 3 year period (August 2001–July 2004) by a prospective surveillance at public and private microbiology laboratories.^14^ Out of 1095 incident cases of candidemia, 138 (12.6%) occurred in adults with HM. The aims of this study were to determine the clinical risk factors for candidemia due to fluconazole-resistant isolates at presentation, and to evaluate whether bloodstream infection with fluconazole-resistant Candida adversely affects clinical outcome and/or is associated with cross-resistance to other triazoles. C.albicans was the most common species (32.7%) followed by C.parapsilosis (19.5%), C.krusei (16.6%), and C.glabrata (12.3%). No C.albicans and C.tropicalis and only one C.parapsilosis isolate was resistant to fluconazole. Almost all C.glabrata and C.krusei isolates were resistant or susceptible-dose -dependent to fluconazole (MIC > 8 mg/L). By multivariate analysis, triazole therapy, gastrointestinal tract surgery in the 30 days before candidaemia and age >65 years were predictive of fluconazole-resistant candidaemia. Thirty day crude mortality was 40%. Fluconazole-resistant isolates were associated with increased risk of mortality by univariate and Kaplan–Meier survival analyses.

### Epidemiology in HSCT recipients:

For several decades, the most relevant epidemiological and clinical data on IFDs in the HSCT setting derived from the studies of the Fred Hutchinson Cancer Research Center (FHCRC) of Seattle. Although these data may not reflect the current epidemiology of Candida infections in the transplant setting they continue to be of great value. A retrospective study on patients undergoing allogeneic HSCT from January 1994 to June 1997, all submitted to prophylaxis with fluconazole, showed that 44% of patients were colonized by Candida but only 4.6% of patients developed candidemia with a candidemia-associated mortality rate of 20%...^13^ *C. albicans* was the most common pretransplant colonizing isolate, whereas resistant species such as *C. krusei* and *C. glabrata wer*e most commonly isolated after transplantation and exposure to fluconazole. Most of strains isolated from blood were resistant to fluconazole, including 2 C.albicans isolates which accounted for only 7% of cases of candidemia. It is likely that fluconazole administration decreased colonization with azole-susceptible organisms, allowing resistant organisms to emerge. This study seems to show that antifungal prophylaxis is associated to low incidence of candidemia and attributable mortality in HSCT recipients despite the emerging phenomenon of colonization and infection by fluconazole resistant strains.

In the last few years, some multicenter studies on IFD in HSCT recipients have been reported from Europe and North America and the main results, with a particular focus on Candida infections, are herein summarized.^20^,^23^,^24^

A retrospective survey conducted in 11 tertiary care centers or university hospitals in Italy between 1999 and 2003 assessed the incidence of IFD in 1979 patients submitted to autologous HSCT and 1249 patients submitted to allogeneic HSCT. Overall, the incidence of IC was 1.1% and 0.8% in the allogeneic and autologous transplant setting, respectively. *C. albicans* was the etiologic agent in 43% of cases and non-albicans species of *Candida* were responsible for the other 57% of cases. Among non-*albicans* species of *Candida*, *C.glabrata, C.krusei and C.guilliermondii* were the most frequently encountered. IC represented the most frequent IFD in autologous HSCT, a condition rarely complicated by filamentous fungi infections, whereas in allogeneic HSCT IA was the most frequent IFD (incidence 6.3%). This large retrospective study showed a low incidence of IC in HSCT patients; the overall rate of mortality due to *Candida* infection was 0.5% but the attributable mortality rate was 44%. No difference in attributable mortality rate emerged among the two transplant procedures. Outcome was poorer among patients with infections due to non-*albicans Candida* strains than among those with *C.albicans* infection, although the difference was not significant (50% vs. 33%; *P*=0.5).

The Transplant Associated Infections Surveillance Network, a network of 23 US transplant centers, prospectively enrolled HSCT recipients with proven and probable IFIs occurring between March 2001 and March 2006.^24^ The denominator data on all HSCTs performed and clinical, diagnostic, and outcome information for each IFD case were collected. Out of 983 IFDs among 875 HSCT recipients 28% were IC. Among 276 patients with invasive candidiasis, *C. glabrata* was the most common organism (33%), followed by *C. albicans* (20%). Median onset of IC after HSCT was 61 days. Within a cohort of 15,820 incidence patients for whom follow-up data were available the overall 12-month cumulative incidence of IFDs was 3.4%. In particular the 6-month and 12-month cumulative incidences for IC were 1.0% and 1.1%, respectively. Overall, one year survival among HSCT patients with IC was 33.6%. No specific data are available in this study regarding the cumulative incidence and survival stratified according to type of transplant. This study seems to confirm other experiences that IC, a relatively common IFD among this patient population during the 1980s and early 1990s, now represents a minority of IFDs and non-*albicans Candida* species accounts for almost most of these infections. Widespread use of azole prophylaxis has likely influenced the decreased incidence and shift in epidemiology in the HSCT setting, although other factors may play a role.

As above mentioned, with use of data from the North American PATH Alliance registry which collected information regarding IFDs, a multicenter, prospective, observational study to assess the epidemiologic characters and outcomes of IFD was performed in HSCT recipients.^20^ Sixteen medical centers reported data on adult HSCT recipients with proven or probable IFD during the period July 2004 through September 2007. Out of 250 proven or probable IFDs, documented in 234 patients submitted to allogeneic (161 patients) or autologous (73 patients) HSCT, Candida species caused 25% of the IFDs in both HSCT populations. Overall, non-albicans Candida species accounted for 75.8% of the isolates and C.glabrata was the most common specie (43.5%) with a similar impact in the allogeneic (46.5%) and autologous (36.8%) transplant setting. Four cases (11.3%) were due to C.krusei. The median interval between HSCT and diagnosis of IC was 28 days in autologous HSCT recipients and 108 days in allogeneic HSCT recipients. Allogeneic transplant recipients who received a myeloablative conditioning regimen were more likely to receive a diagnosis of IC early after HSCT (median interval, 65 days), compared with those who received a nonmyeloablative conditioning regimen (median interval, 590 days). Most of patients with IC were treated with caspofungin (52.6%) whereas 35% and 31.6% of patients received a lipid formulation of amphotericin B and fluconazole, respectively. The majority of HSCT recipients with IC (67.7%) were reported to have responded (completely or partially) to the administered therapy. The 12-week mortality rate in patients with IC was 48.9%, higher than that observed in patients with IA (35.5%). The true attributable mortality rate was unclear in this registry. Because of the small number of patients with IC in this study, the authors were not able to obtain data on potential risk factors associated with mortality in HSCT recipients with IC

## Current Therapeutic Options of Invasive Candidiasis in HM and HSCT Patients:

In the last years, several prospective, controlled studies in the prophylaxis of IFDs have been conducted in both the HM and HSCT setting. On the contrary, most of the prospective controlled trials that demonstrated the efficacy of antifungal drugs in the treatment of candidemia and invasive candidiasis focused on nonneutropenic patients not affected by an HM. For these reasons, international guidelines give specific indications for the prophylaxis strategies in HM and HSCT patients, whereas the recommendations on therapy of documented infections based on the results observed in the general population should be considered with caution.

### Prophylaxis:

Prevention strategies for IFD in HM and HSCT patients are based on environmental precautions and antimicrobial prophylaxis. Although there is general agreement with respect to the environmental precautions, the role of pharmacological prophylaxis is still debated. Until few years ago fluconazole, and to a less extent itraconazole, were recommended for primary prophylaxis against *Candida* infection in neutropenic patients and allogeneic HSCT recipients.^29^–^33^ This prophylactic strategy proved to decrease the rate of *Candida* infection and, in the transplant setting, was associated with an overall survival benefit at long-term follow-up.^31^ However, a major limitation of a *Candida* oriented prophylaxis is the lack of activity against moulds which represent the most frequent cause of IFDs in such populations. In the past few years, several broad spectrum antifungal drugs have been randomly compared with standard fluconazole for the prophylaxis of IFD in acute leukaemia and HSCT, with the aim to prevent both Candida and mould infections. The data of recent, large, randomized trials of antifungal prophylaxis in HM and HSCT patients, with particular attention to the prevention of IC, are summarized in table 1.^34^–^37^ The new drugs posaconazole, voriconazole and micafungin proved to be as effective as fluconazole in the prevention of IC in high risk patients with neutropenia or graft versus host disease. The rate of IC in these studies was particularly low and there was no significant emergence of triazole resistant Candida strains.

According to the results of the above studies, international guidelines consider fluconazole (400 mg/d i.v or p.o) as the drug of choice for primary prophylaxis in allogeneic HSCT recipients in the early phases from transplant.^38^–^41^ Posaconazole (200 mg/tid p.o), which is as effective as fluconazole in the prevention of Candida infections but more effective in the prevention of mould infections, is the drug of choice in patients with severe GVHD. In patients with acute myeloid leukemia undergoing intensive chemotherapy fluconazole is no more the drug of choice but posaconazole is now recommended. A potential limitation of posaconazole is represented by the erratic oral absorption, especially in patients with chemotherapy related intestinal mucositis, intestinal GVHD and/or diarrhea. In this setting, monitoring of drug levels during therapy should be considered. The role of voriconazole in the transplant setting deserves further evaluation. It should be stressed that for the strict prevention of *Candida* infection no new antifungal drug showed any advantage compared to fluconazole.

### Therapy of invasive candidiasis:

All echinocandins (caspofungin, micafungin and anidulafungin) and voriconazole have been evaluated in controlled clinical trials in the treatment of candidemia.^42^–^47^ The data of the overall population enrolled and specifically those of patients with HM and HSCT recipients are detailed in Table 2. All new antifungal drugs proved to be effective, in some cases more effective, as compared to fluconazole or formulations of amphotericin B in the treatment of candidemia. Very limited evaluation of efficacy was possible in the few patients with neutropenia, HM or submitted to HSCT.

In view of these data, the most recent invasive candidiasis treatment guidelines^38^–^40^,^48^ provided recommendations for the general population and extended some indication for neutropenic patients even in absence of specific evidence. Fluconazole (loading dose of 800mg [12 mg/kg], then 400 mg [6 mg/kg] daily) is considered the primary treatment for nonneutropenic patients with mild-to-moderate candidemia and no recent azole exposure. An echinocandin antifungal is recommended as primary therapy for nonneutropenic and neutropenic patients with moderately severe or severe candidemia or those who have had recent azole exposure. Transition to oral fluconazole is appropriate once the patient is clinically stable and the isolate is definitely speciated or susceptibility to fluconazole is confirmed. Fluconazole is a reasonable alternative to echinocandin therapy in neutropenic patients with mild-to-moderate candidemia.

## Conclusions:

Invasive *Candida* infections no more represent a major complication of patients with HM submitted to intensive chemotherapy and of HSCT recipients. All retrospective and prospective surveys and clinical trials in such populations seem to demonstrate that a prophylaxis strategy with the first generation triazoles (fluconazole and itraconazole), the second generation triazoles (posaconazole and voriconazole) and the echinocandins (caspofungin, micafungin and anidulafungin) are effective in the prevention of IC. Fluconazole may be chosen as the drug of choice considering its efficacy and the high costs of the other antifungal drugs, but the emerging epidemiological and clinical impact of IA and of other filamentous fungi infections in these populations has led to prefer broad spectrum, mould active drugs in several clinical settings.

Although the incidence of IC appears to be quite low in HM and HSCT patients its high mortality rate continues to be a crucial problem despite the availability of new effective antifungal drugs. On the other hand, it should be considered that the poor outcome of patients with IC most likely reflects the underlying compromised immune status and organ function of patients who develop *Candida* infection.

## Figures and Tables

**Table 1:** Recent controlled studies of antifungal prophylaxis in patients with HM and HSCT recipients: efficacy in the prevention of invasive candidiasis.

***Author, year of publication (reference n. )***	***Population***	***Antifungal drugs***	***Incidence of invasive Candida infections***
*Cornely, 2007 (34)*	*Neutropenic patients with acute myeloid leukemia and myelodisplasticsyndromes*	*Posaconazole vs fluconazole or itraconazole*	*3/304(1%) vs 2/298 (0.7%)*
*van Buric,2004 (35)*	*Allogeneic HSCT recipients until engraftment or day 42 post transplant*	*Micafungin vs fluconazole*	*4/425(0.9%) vs 2/457(0.4%)*
*Ullmann, 2007 (36)*	*Allogeneic HSCT recipients with graft versus host disease*	*Posaconazole vs fluconazole*	*4/301(1.3%) vs 4/299(1.3%)*
*Wingard, 2010 (37)*	*Allogeneic HSCT recipients until day 100 or 180 post transplant*	*Voriconazole vs fluconazole*	*3/305 (1%) vs 3/295 (1%)*

**Table 2:** Recent controlled studies of therapy of invasive candidiasis. Data of the overall population and available data of patients with HM and HSCT recipients.

***Author, year of publication (reference n. )***	***Antifungal drugs***	***Success rate at end of study drug administration in the overall population***	***Available data in patients with HM and HSCT recipients***
*Mora-Duarte, 2002 (42)*	*Caspofungin vs amphotericin B*	*73% vs 62%*	*Success rate in neutropenic patients: 7/14 (50%) vs 4/10 (40%)*
*Betts, 2009 (43)*	*Caspofungin 70/50 mg vs caspofungin 150 mg*	*71.6% vs 68.2%*	*Success rate in neutropenic patients: 2/6 (33%) vs 4/7 (57%)*
*Reboli, 2007 (44)*	*Anidulafungin vs fluconazole*	*74% vs 56.8%*	*Total neutropenic patients: 3 vs 2 cases* *No specific evaluation of success rate*
*Kuse, 2007 (45)*	*Micafungin vs liposomal amphotericin B*	*89.6% vs 89.5%*	*Success rate in neutropenic patients: 18/24 (75%) vs 12/15 (80%)*
*Pappas, 2007 (46)*	*Micafungin 100 mg vs micafungin 150 mg vs caspofungin 50 mg*	*76.4% vs 71.4% vs 72.3%*	*Total neutropenic patients: 22 vs 17 vs 11 cases* *Total HSCT recipients:10 vs 4 vs 4 patients* *No specific evaluation of success rate*
*Kullberg, 2005 (47)*	*Voriconazole vs amphotericin B followed by fluconazole*	*70% vs 74%*	*Neutropenic patients were excluded from the study*
